# Norovirus-specific monoclonal antibodies that block histo-blood group antigen binding isolated from healthy donors

**DOI:** 10.1128/jvi.00497-26

**Published:** 2026-05-26

**Authors:** Sabina E. W. Leonard, Olivia C. Powers, Parastoo Amlashi, Mark von Itzstein, Perry T. Wasdin, Toma M. Marinov, Alexandra A. Abu-Shmais, Ivelin S. Georgiev, Grant S. Hansman

**Affiliations:** 1 Vanderbilt Center for Antibody Therapeutics, Vanderbilt University Medical Center 12328, Nashville, Tennessee, USA; 2 Department of Pathology, Microbiology and Immunology, Vanderbilt University Medical Center 12328, Nashville, Tennessee, USA; 3 Institute for Biomedicine and Glycomics, Griffith University 265012, Gold Coast, Queensland, Australia; 4 Center for Computational Microbiology and Immunology, Vanderbilt University Medical Center 12328, Nashville, Tennessee, USA; 5 Vaccine Research Center, National Institute of Allergy and Infectious Diseases, Bethesda, Maryland, USA; 6 Vanderbilt Institute for Infection, Immunology and Inflammation, Vanderbilt University Medical Center 12328, Nashville, Tennessee, USA; 7 Department of Computer Science, Vanderbilt University 5718, Nashville, Tennessee, USA; 8 Center for Structural Biology, Vanderbilt University 5718, Nashville, Tennessee, USA; 9 Department of Biomedical Informatics, Vanderbilt University Medical Center 12328, Nashville, Tennessee, USA; 10 Program in Chemical and Physical Biology, Vanderbilt University Medical Center 12328, Nashville, Tennessee, USA; 11 Department of Chemical and Biomolecular Engineering, Vanderbilt University 5718, Nashville, Tennessee, USA; 12 Department of Biochemistry, Vanderbilt University School of Medicine 12327 , Nashville, Tennessee, USA; University of Michigan Medical School, Ann Arbor, Michigan, USA

**Keywords:** histo-blood group antigen, antibody, norovirus

## Abstract

Human noroviruses are a leading cause of acute gastroenteritis, yet no licensed vaccine or antiviral is currently available. The extensive genetic diversity of noroviruses and the limited cross-protective immunity between genotypes complicate the development of broadly effective interventions. To systematically characterize norovirus-specific B cell responses from healthy individuals, we applied linking B cell receptor to antigen specificity through sequencing (LIBRA-seq) to peripheral blood mononuclear cells using a genetically diverse panel of norovirus capsid protruding (P) domain antigens. Antigen-specific B cells were identified at single-cell resolution and selected for recombinant monoclonal antibody (mAb) expression. A total of 25 norovirus-specific mAbs were generated and evaluated for binding breadth across multiple genogroup II (GII) genotypes. Binding analyses revealed substantial heterogeneity, with some mAbs displaying narrow, genotype-restricted recognition, whereas others exhibited broad cross-reactivity. Epitope competition assays using a subset of mAbs demonstrated both overlapping and distinct binding sites on the P domain. Functional activity, assessed using a surrogate neutralization assay measuring inhibition of histo-blood group antigen (HBGA) binding, showed that several broadly reactive antibodies inhibited virus-like particle attachment to HBGAs with variable potency across genotypes, whereas others exhibited genotype-restricted inhibition. Together, these results demonstrate that LIBRA-seq enables efficient mapping of human antibody repertoires to norovirus at the population level and reveals that natural exposure generates a diverse B-cell repertoire with varying specificity and functional breadth.

**IMPORTANCE:**

Human noroviruses are the leading cause of acute viral gastroenteritis worldwide, yet individuals experience repeated infections throughout life, indicating that protective immunity is incomplete and remains poorly understood. In this study, we applied the high-throughput LIBRA-seq approach to systematically map the norovirus-specific antibody repertoire in healthy human donors, enabling the identification of monoclonal antibodies that recognize and functionally inhibit clinically relevant genotypes. This work provides new insight into the breadth and specificity of naturally acquired humoral immunity by establishing a comprehensive framework for analyzing antigen-specific B cell responses in the general population and generates a well-defined panel of human antibodies that will be useful for future studies of norovirus immunity, vaccine evaluation, and therapeutic development.

## INTRODUCTION

Human noroviruses are the leading cause of acute gastroenteritis worldwide (1). Despite this public health importance, no licensed vaccine, antiviral, or targeted therapy exists, and current management is limited to supportive care, primarily oral rehydration and electrolyte replacement (2). A major challenge in developing effective interventions is the extraordinary genetic and antigenic diversity of human noroviruses. Human norovirus has a single-stranded, positive-sense RNA genome encoding non-structural and structural proteins, with three open reading frames (ORFs) (3). Noroviruses are grouped into 10 genogroups (GI–GX), which are subsequently subdivided into numerous genotypes (4). Human norovirus capsid gene (ORF2) frequently evolves into genetic and antigenic variants (5–7), and the GII genotype 4 (GII.4) variants have dominated over the past few decades with approximately 5% capsid amino acid divergence from previous variants (5, 6). This continual evolution complicates vaccine design and therapeutic development. Moreover, short- and long-term protection to norovirus reinfections varies greatly among individuals (8–12). Therefore, understanding the patterns of antibody cross-reactivity and/or histo-blood group antigen (HBGA) blocking across diverse norovirus genotypes in the general population is critical for the development of broadly protective interventions.

Expression of the capsid gene in insect cells results in the formation of virus-like particles (VLPs) that are morphologically and antigenically similar to native virions (13). Structural studies of the VLP revealed two distinct domains: the shell (S) domain, which encases the viral RNA, and the protruding (P) domain, which contains the determinants for co-factor binding and antibody recognition (13). The P domain can also be expressed in *Escherichia coli*, where it forms biologically relevant P dimers that closely mimic the P domain on VLPs and have been extensively used for structural and functional studies (14–17). Human norovirus capsids bind to HBGA co-factors, a critical interaction for viral infection (18–23). Across the ABH- and Lewis-HBGA types, at least nine distinct HBGAs have been shown to interact with human noroviruses. Most GII genotypes engage HBGAs at a common site on the P domain, making this site a central target for vaccine and therapeutic development aimed at blocking viral attachment and neutralizing infection (24).

Recent studies have characterized norovirus capsid-specific monoclonal antibodies (mAbs) that can inhibit norovirus VLPs from binding to HBGAs and/or inhibit norovirus virion replication in cell culture (25–31). Most of the HBGA blocking antibodies analyzed (as a surrogate for neutralization) were genotype specific with limited cross-reactivity, whereas several mAbs were shown to be broadly reactive and blocked HBGA binding across GI genotypes (29) and GII genotypes (31). Interestingly, one human norovirus IgA isolated from a norovirus-infected patient bound to the side of the P domain and led to virion aggregation and neutralization in cell culture (25). We also showed that one neutralizing mAb partially blocked the HBGA pocket, but mainly engaged variable residues surrounding the HBGA-binding site on the top of the P domain (30). In addition to mAb discovery, we and several other groups have also developed norovirus nanobodies that target the capsid protein and have identified nanobodies that directly and indirectly block VLP binding to HBGAs and/or neutralize norovirus in cell culture (32–39). However, norovirus nanobodies that directly block the HBGA pocket were genotype specific, whereas the broadly reactive nanobodies bound to highly conserved regions on the lower region of the P domain.

Despite these advances, there remains a limited understanding of the human B cell repertoire against noroviruses at a population level. Recent technological innovations, including linking B cell receptor to antigen specificity through sequencing (LIBRA-seq), enable high-throughput mapping of B cell receptor sequences to antigen specificity, facilitating the precise determination of cross-reactivity and neutralization profiles (40, 41). This information can advance our knowledge of possible host protection in the general population and likely long-term immunity (or lack thereof) against reinfections.

In this study, we used LIBRA-seq to characterize 25 unique norovirus-specific mAbs from 10 healthy human blood donors, facilitating a novel and systematic approach to map the human norovirus B cell repertoire. The mAbs were evaluated for cross-reactivity and HBGA-blocking activity, revealing several antibodies with potent inhibition against the globally dominant GII.4 and emerging GII.17 strains. These results improve our understanding of naturally acquired norovirus immunity and define patterns of antibody recognition across multiple genotypes, providing a framework for future studies aimed at identifying conserved epitopes relevant to vaccine and antiviral development.

## RESULTS

### Isolation of norovirus-specific mAbs using LIBRA-seq

**Fig 1:**
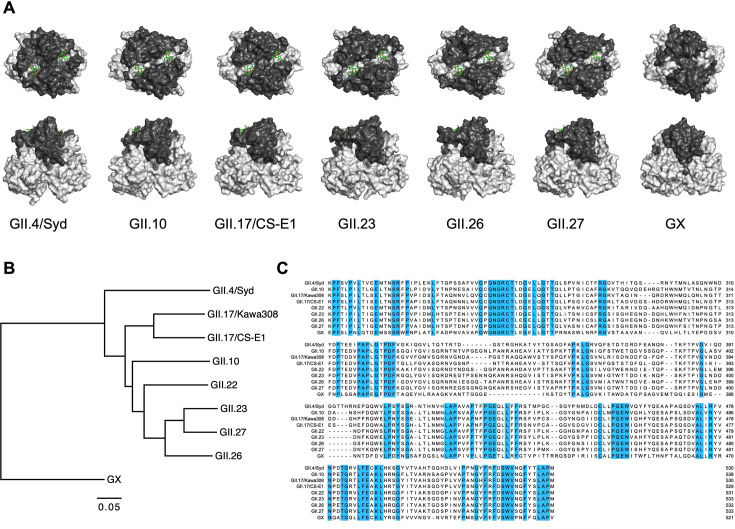
Norovirus P domains used with LIBRA-seq. (**A**) The X-ray crystal structures of the norovirus P domains used in this study were previously determined GII.4 (4OOS), GII.10 (3ONU), GII.17/CS-E1 (8V97), GII.23 (9EDN), GII.26 (8V99), GII.27 (9EDO), and GX (9EDQ). The P domains were colored according to the P1 subdomain (gray) and P2 subdomain (black), showing the top and side views. The HBGA A-trisaccharide (green) from the GII.10 A-trisaccharide complex (3PA1) was modeled into the human norovirus P domains to show the common HBGA pocket and has been determined for GII.4 (4WZT), GII.10, GII.17 (5LKC), and GII.27 (9OLT). (**B**) A phylogenetic tree of the norovirus P-domain amino acid sequences was generated to illustrate genetic diversity, with GX included as an outgroup. Sequences were aligned using the Clustal Omega (44) multiple sequence alignment server (https://www.ebi.ac.uk/jdispatcher/msa/clustalo) (45) with default settings, and gaps were removed from the alignment prior to tree generation (44, 45). The scale corresponds to substitutions per site. (**C**) Amino acid alignment of the norovirus P domains and VLPs (GII.4, GII.10, and GII.17/Kawa308) showing patches of conserved regions (highlighted blue). Pairwise comparison of the deposited P-domain amino-acid sequences showed that the human norovirus P domains shared approximately 60%–87% amino-acid identity, whereas the bat GX P domain shared lower identity (~47%–50**%**) with the human norovirus P domains.

To identify norovirus-specific antibodies, we used LIBRA-seq on peripheral blood mononuclear cells (PBMCs) from healthy human donors with an antigen screening library that included genetically and antigenically diverse (4, 38, 39, 42, 43) norovirus P domains (Fig. 1). LIBRA-seq is a high-throughput single-cell sequencing technology that identifies antigen-specific B cells by conjugating antigens to unique oligonucleotide barcodes, therefore enabling the screening of a given B cell repertoire simultaneously against a large number of antigens (40, 41). LIBRA-seq therefore allows high-throughput identification and prioritization of B cells with diverse antigen specificity profiles, including virus-specific and cross-reactive B cells. Following computational analysis, LIBRA-seq provides paired heavy and light chain sequences together with norovirus antigen specificity, reported as a LIBRA-seq score (LSS). The LSS reflects the relative strength of binding between a B cell and a given norovirus antigen. For this study, an LSS > 2 was used as the threshold for defining a positive signal for norovirus antigen recognition. The norovirus antigen panel included seven structurally characterized human norovirus P domain genotypes (GII.4, GII.10, GII.17, GII.22, GII.23, GII.26, and GII.27), and one bat norovirus P domain genotype (GX; Fig. 1).

**Fig 2:**
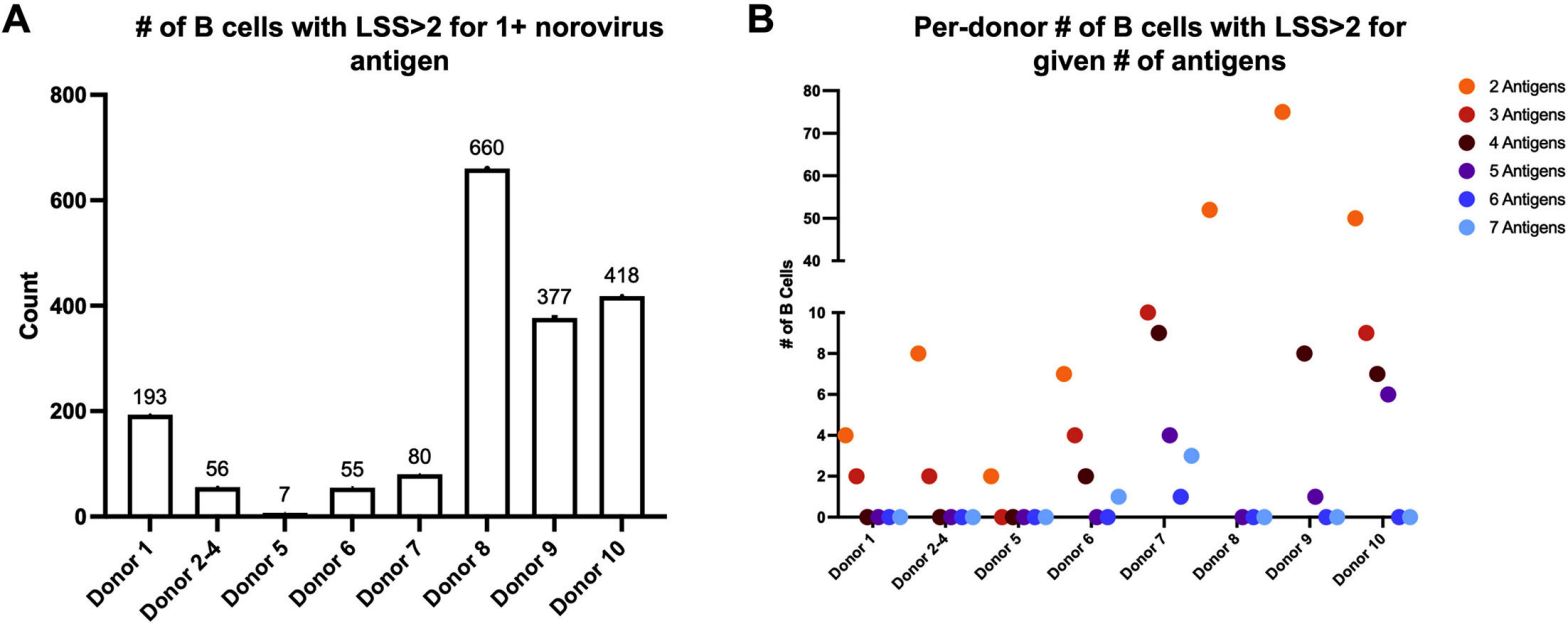
Identification of norovirus-specific antibodies using LIBRA-seq. (**A**) Number of B cells with an LSS > 2 for at least one norovirus antigen per donor, noting that donors 2, 3, and 4 had low cell numbers and their PBMCs were pooled. (**B**) Distribution of B cell antigen specificity by donor, grouped by the number of norovirus antigens recognized (2–7). Each dot represents a cell population with a defined antigen specificity count.

Eight sorting experiments were performed using PBMCs from 10 donors (Fig. 2A; Fig. S1). PBMCs from donors 2, 3, and 4 were combined into a single sample prior to sorting due to low cell numbers, whereas all other donors were screened as individual samples, for a total of eight LIBRA-seq experiments. The number of norovirus-specific B cells recovered (LSS > 2 for at least one norovirus antigen) varied across donors, ranging from 7 cells in donor 5 to 660 cells in donor 8 (Fig. 2A). Across all donors, the mean number of norovirus antigen-specific B cells was approximately 230 (standard deviation ≈ 231). Predicted binding breadth also varied among donors, but all donors had at least two B cells predicted to bind two or more norovirus antigens (Fig. 2B). Donors 6 and 7 each had at least one B cell predicted to bind all seven norovirus antigens, while several donors had cells predicted to bind five or more norovirus antigens (donors 9 and 10).

For antibody expression, we prioritized B cells with high LSS values for two or more norovirus antigens and low reactivity to the negative control antigen (HIV envelope protein), as these were most likely to represent cross-reactive or cross-neutralizing antibodies. Additional selection criteria included isotype, inferred germline gene usage, level of somatic hypermutation, and complementarity-determining region (CDR) length to ensure diversity among antibody lineages (Table 1). Using these criteria, 25 B cells were selected for recombinant mAb expression as human IgG1.

**TABLE 1:** Sequence features of lead antibodies identified from LIBRA-seq^*a*^

Donor ID	Antibody ID	Native isotype	IGHV gene	IGHV identity	CDRH3 length	IGL(K)V gene	IGL(K)V identity	CDRL3 length
2	343	IGHG2	IGHV3-7	0.93	11	IGLV2-11	0.96	10
2	347	IGHG2	IGHV3-15	0.85	12	IGKV4-1	0.87	9
2	96	IGHA1	IGHV1-69	0.96	12	IGLV2-14	0.94	9
2	273	IGHA2	IGHV3-21	0.94	14	IGKV3-20	0.97	9
2	107	IGHG1	IGHV4-NL1	0.97	19	IGKV3-11	0.98	8
1	53	IGHG1	IGHV1-69	0.88	15	IGKV1-9	0.97	9
1	1041	IGHG1	IGHV1-69	0.97	17	IGKV4-1	0.98	9
1	1064	IGHA1	IGHV3-48	0.97	16	IGKV1-5	0.99	9
1	1977	IGHA1	IGHV3-21	0.95	19	IGLV2-14	0.97	10
1	2301	IGHA1	IGHV3-23	0.90	11	IGLV2-14	0.92	11
1	3024	IGHA1	IGHV3-23	0.93	16	IGLV2-14	0.97	11
1	3029	IGHG2	IGHV5-51	0.96	17	IGKV3-15	0.99	10
1	3048	IGHA1	IGHV3-23	0.94	19	IGLV2-14	0.97	11
4	471	IGHG1	IGHV3-23	0.87	14	IGKV1D-39	0.91	8
4	664	IGHG1	IGHV4-30-2	0.94	13	IGLV1-51	0.97	11
4	1444	IGHG1	IGHV1-69	0.91	15	IGKV3-11	0.95	11
5	2837	IGHG1	IGHV3-23	0.88	19	IGLV2-14	0.94	10
7	5656	IGHG1	IGHV3-23	0.87	15	IGKV1D-39	0.93	9
8	82	IGHG1	IGHV4-34	0.81	23	IGLV2-8	0.93	11
8	734	IGHA1	IGHV1-69	0.88	19	IGLV2-8	0.94	10
8	2254	IGHG1	IGHV4-39	0.87	32	IGKV1D-39	0.94	8
8	2579	IGHA1	IGHV1-69	0.88	19	IGLV2-8	0.97	10
8	3552	IGHG1	IGHV1-2	0.90	25	IGKV2-28	0.95	9
8	6548	IGHG1	IGHV4-34	0.97	18	IGKV3-20	0.97	11
8	9152	IGHG1	IGHV4-59	0.90	18	IGKV3-20	0.95	9

^*a*^ Antibodies were of multiple native isotypes, utilized unique inferred VH and VL genes, had varying degrees of somatic hypermutation and CDRH/L3 lengths.

### MAb binding to norovirus P domains

**Fig 3:**
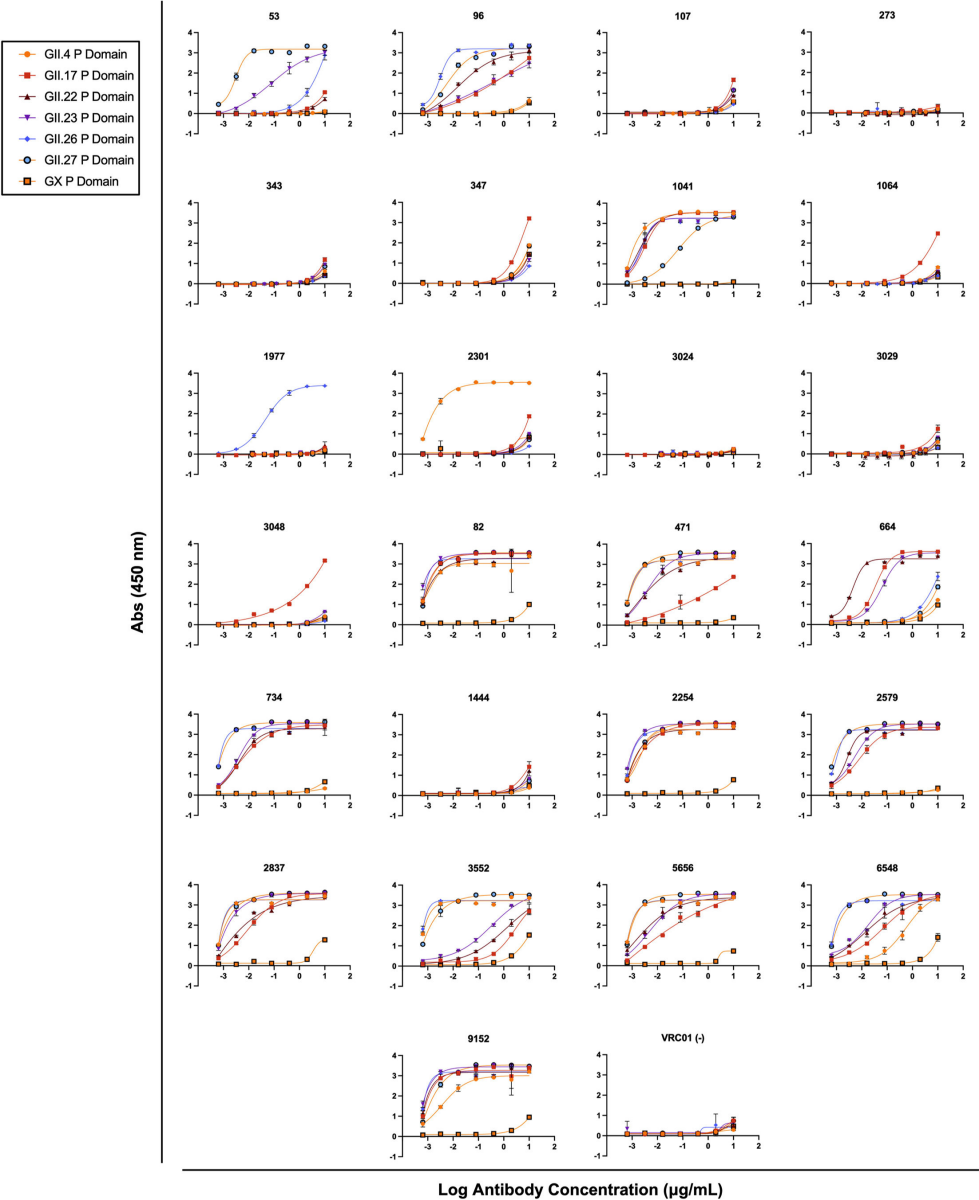
ELISA curves of mAb binding to the P domains. MAbs were screened in duplicate wells from a starting concentration of 10 µg/mL and fivefold serially diluted. The binding absorbance was measured at OD_450_. Error bars are shown for duplicated wells, and the norovirus P domains are colored accordingly.

**Fig 4:**
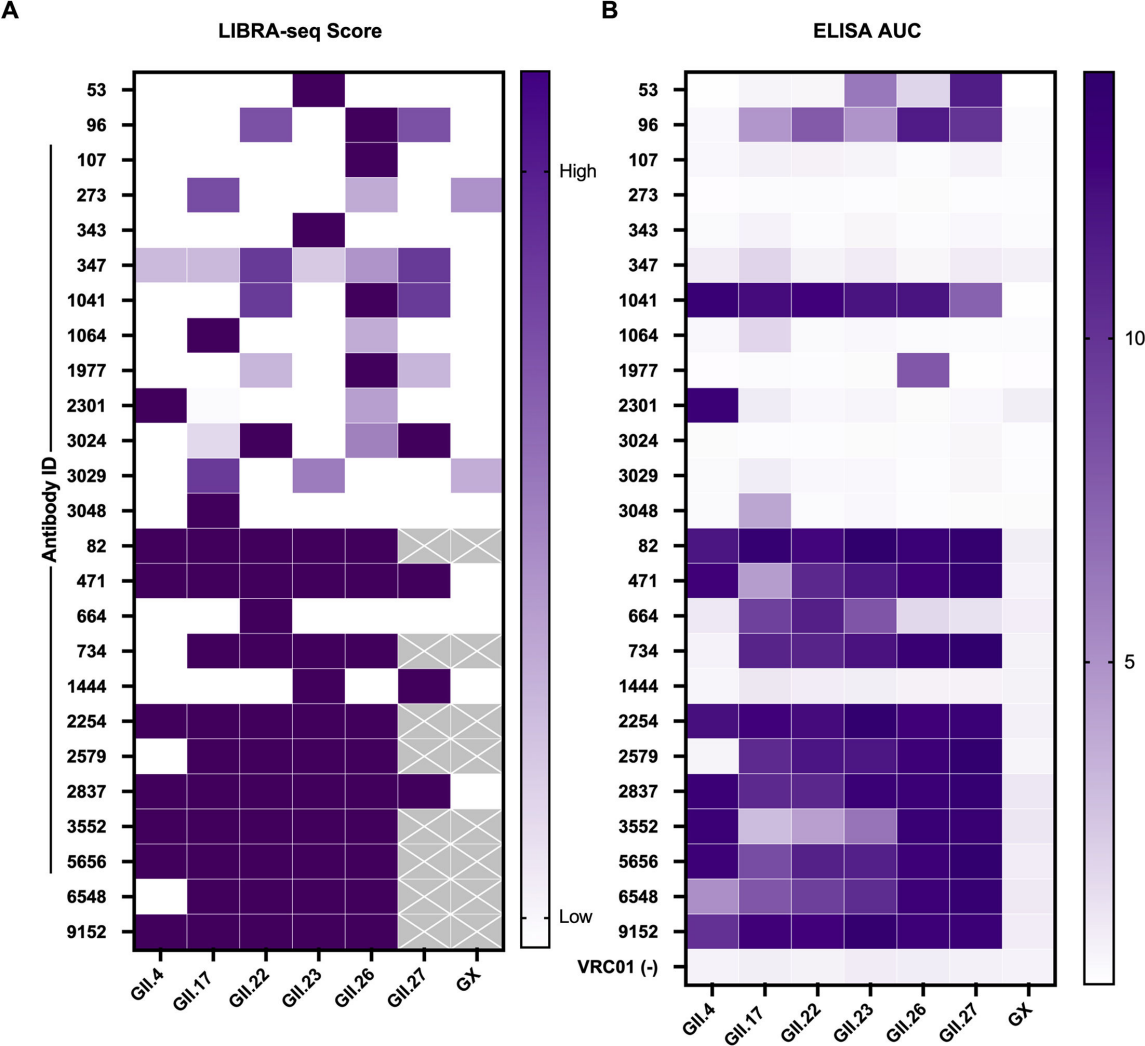
MAb candidates demonstrate broad binding to norovirus P domains. (**A**) LIBRA-seq score values, where dark purple indicates the highest LIBRA-seq score (predicted binding), and white shows the lowest LIBRA-seq score. Gray boxes indicate that corresponding antigen was not included in the respective LIBRA-seq experiment. LIBRA-seq reactivity is shown as relative signal (high/low) because scores were normalized within each donor sort and are not directly comparable across different sorts. (**B**) ELISA area under the curve (AUC) values, where dark purple indicates the highest AUC (greater binding via ELISA), and white shows the lowest AUC. A minimal binding interaction between the P domain and mAb was cut off at an ELISA AUC value of 4 or greater. The data shown are a single representative biological replicate.

A total of 25 mAbs were screened by direct enzyme-linked immunosorbent assay (ELISA) for binding to GII.4, GII.17, GII.22, GII.23, GII.26, GII.27, and GX norovirus P domains. Most mAbs bound the human norovirus P domains in a dose-dependent manner, whereas all mAbs showed poor binding to the bat norovirus P domain (Fig. 3). The resulting binding curves revealed substantial heterogeneity in mAb binding profiles across the different human norovirus genotypes. LSS and ELISA area under the curve (AUC) values, calculated from curves shown in Fig. 3, were used to quantify and compare mAb-P domain interactions, with darker purple indicating higher LSS (predicted binding) and higher AUC values (stronger ELISA binding), and white indicating minimal or no binding (Fig. 4). An ELISA AUC threshold of 4 was set, as this value identifies strong binders and provides clear separation from background signal. Across the entire mAb panel, good agreement was observed between LIBRA-seq predictions and ELISA measurements. With a positive LSS defined as >2 and positive ELISA binding defined as AUC > 4, we observed an overall 5.6% false positive rate (LSS > 2, AUC < 4 for a given norovirus antigen) and an 8.8% false negative rate (LSS < 2, AUC > 4 for a given norovirus antigen; Fig. 4). Antibody-antigen pairs that did not have an LSS for a given norovirus antigen were not included in this analysis (Fig. 4). Although minor discrepancies were noted for several mAb-P domain pairs (e.g., mAbs 96, 664, and 1444), the overall concordance supported the use of LIBRA-seq as a reliable high-throughput approach for prioritizing antigen-specific mAbs prior to downstream validation. Based on an ELISA AUC cut-off value (4 and above), seven mAbs (1041, 82, 471, 2254, 2837, 5656, 6548, and 9152) demonstrated the broadest reactivity, binding all six human GII genotypes tested (Fig. 4B). Four mAbs bound five genotypes, mAb 96 (GII.17/GII.22/GII.23/GII.26/GII.27), mAb 734 (GII.17/GII.22/GII.23/GII.26/GII.27), mAb 2579 (GII.17/GII.22/GII.23/GII.26/GII.27), and mAb 3352 (GII.4/GII.22/GII.23/GII.26/GII.27). One mAb bound three genotypes, mAb 664 (GII.17/GII.22/GII.23). One mAb bound two genotypes, mAb 53 (GII.23/GII.27). Three mAbs bound a single genotype, mAb 1977 (GII.26), mAb 2031 (GII.4), and mAb 3048 (GII.17). Of the mAbs tested, 17/25 (68%) bound at least one genotype (Fig. 4B). Overall, these data indicated that genotype-specific and broadly reactive mAbs were successfully isolated from healthy donors using the LIBRA-seq technology.

### Analysis of common mAb binding epitopes on the norovirus P domains

**Fig 5:**
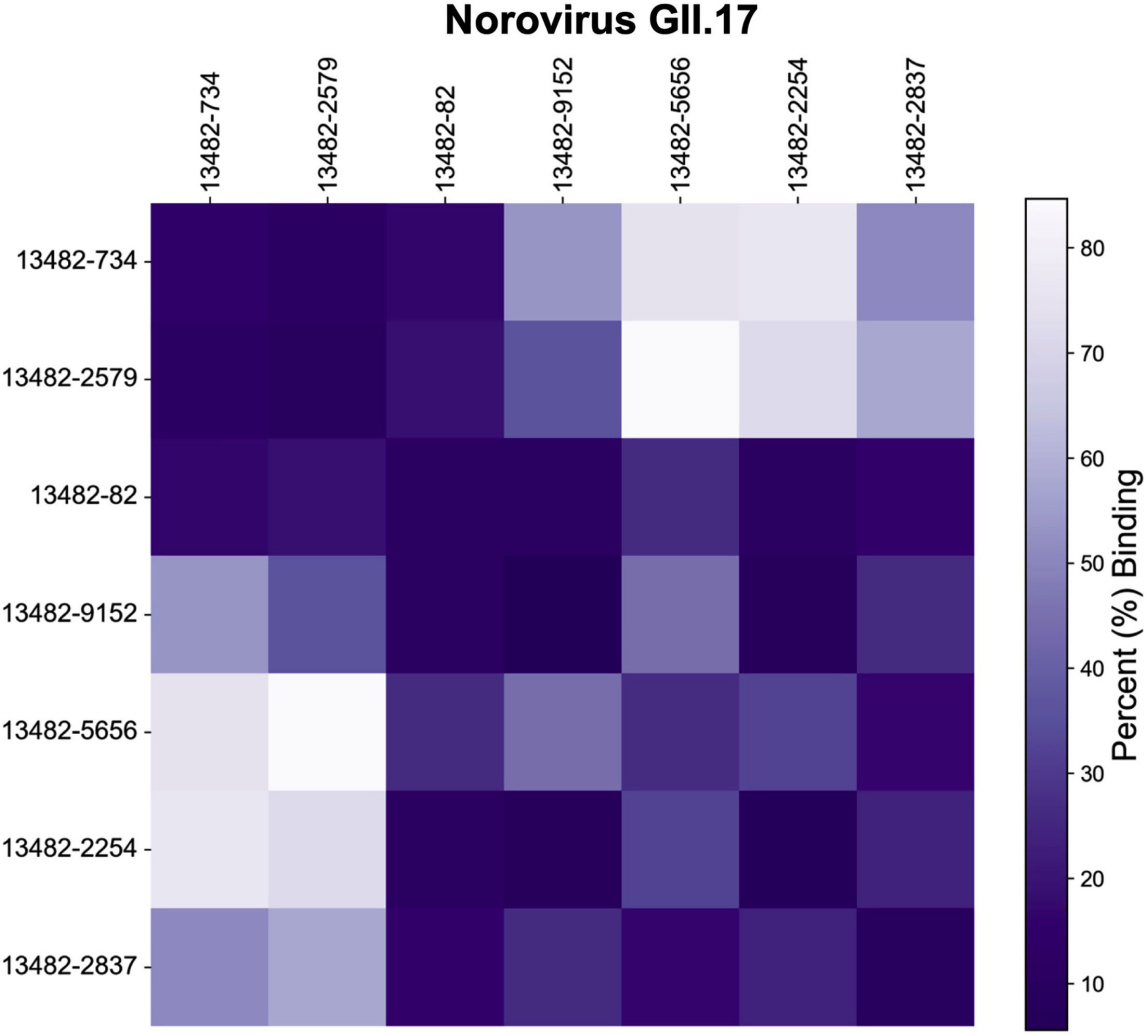
Antibody-antibody competition matrix by ELISA binding. Percent residual binding in the presence of a competitor antibody is shown, with dark purple indicating strong competition between the given pair of antibodies, i.e., antibodies targeting overlapping epitopes on GII.17 P domain.

To determine whether cross-reactive mAbs recognized distinct or overlapping epitopes on the norovirus P domain, a subset of broadly reactive antibodies was evaluated by competition ELISA using the GII.17 P domain as the target antigen (Fig. 5). The antibodies chosen were all classified as strong binders to GII.17 (AUC > 8.5). In this assay, mAbs were classified as competing, consistent with recognition of shared or overlapping epitopes or steric hindrance, if the presence of a test antibody reduced binding of the reference antibody to less than 40% of its maximal signal. While observing competition suggests a shared epitope, an alternate possibility is that binding of the first mAb causes conformational changes to the P domain, preventing binding of the second mAb. Conversely, mAbs were classified as non-competing, indicative of distinct epitopes, if residual binding remained greater than 70%. Competition ELISA revealed multiple mAbs that strongly inhibited reference antibody binding, indicating recognition of a shared or spatially proximal epitope on the GII.17 P domain. Notably, mAb pairs 734/2579, 2254/9152, and 2837/5656 consistently reduced binding below the competition threshold, suggesting that these antibodies target a common antigenic site or sterically interfere with one another’s binding. MAb 82 competed broadly with all antibodies tested. Together, these data indicate that these isolated mAbs could be grouped into three discrete epitope classes.

### Surrogate neutralization HBGA blocking assay

**Fig 6:**
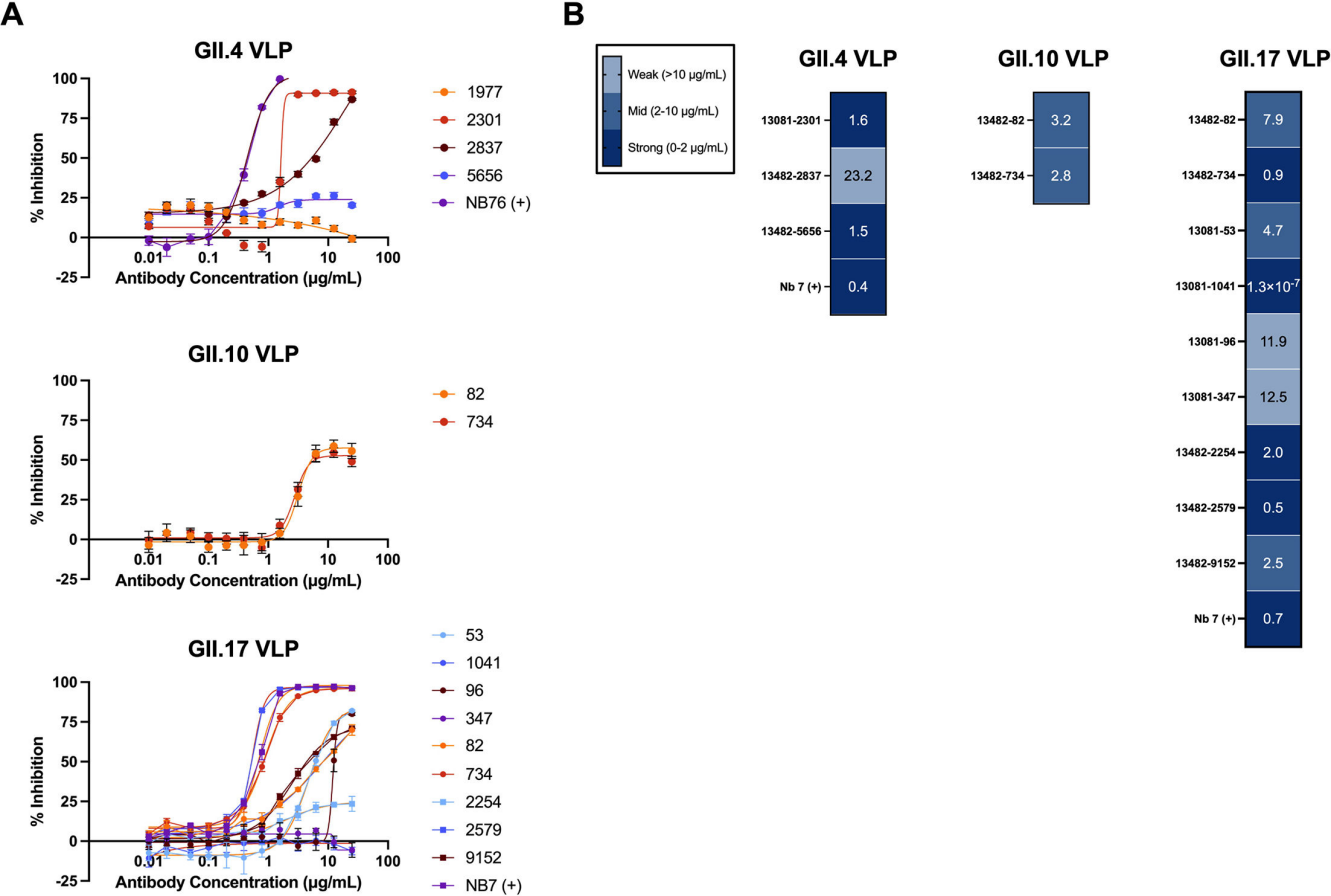
HBGA blocking assay using VLPs and human saliva as a source of HBGAs. (**A**) HBGA blocking curves for mAbs tested against GII.4, GII.10, and GII.17 (Kawa-308) VLPs. Only mAbs that demonstrated HBGA blocking are shown. (**B**) Quantification of values of the HBGA blocking, where IC_50_ values were calculated and labeled as weak (>10 µg/mL), mid (2-10 µg/mL), or strong (<2 µg/mL) for each respective VLP. 76^-GII.4-specific^ and NB7^-GII.17-specific^ directly block the HBGA pocket and were used as positive controls (46).

MAbs that showed detectable binding to a given genotype in ELISA were subsequently tested for HBGA blocking against the same genotype using a surrogate HBGA blocking assay with GII.4, GII.10, and GII.17 (Kawasaki 308) VLPs (Fig. 6A). MAbs that did not show ELISA binding were excluded from the blocking assay for that genotype. HBGA blocking potency varied substantially across the mAb panel and among genotypes (Fig. 6A). Two mAbs (2301 and 2837) exhibited strong HBGA blocking against GII.4 VLPs, with IC₅₀ values below 2 µg/mL, comparable to the positive control Nanobody NB76 (that directly blocks the GII.4 HBGA pocket) (46), indicating efficient blockade of HBGA binding. Against GII.10 VLPs, two mAbs (82 and 734) displayed moderate HBGA blocking activity, with IC₅₀ values of 3.2 and 2.8 µg/mL, respectively (Fig. 6B). Notably, both mAbs also neutralized GII.17 VLPs, with IC₅₀ values of 7.9 µg/mL and 0.9 µg/mL for mAbs 82 and 734, respectively, indicating cross-genotype neutralizing activity (Fig. 6B). In addition to these two mAbs, seven other mAbs (53, 96, 347, 1041, 2254, 2579, and 9152) exhibited varying degrees of HBGA blocking against GII.17 VLPs, comparable to the positive control nanobody NB7 (that directly blocks the GII.17 HBGA pocket) (46). Collectively, these data indicate that although HBGA blocking against GII.10 was limited, the mAbs that were capable of blocking HBGA binding to GII.10 VLPs and also displayed cross-reactive HBGA blocking activity against GII.17 VLPs. These findings highlight genotype-dependent differences in susceptibility to antibody-mediated HBGA blocking.

Comparison with the previous binding analyses revealed that HBGA blocking largely correlated with ELISA and LIBRA-seq data. Broadly binding mAbs, such as mAbs 82 and 734, which displayed high LIBRA-seq scores and elevated ELISA AUC values across multiple P domain genotypes, also exhibited cross-genotype HBGA blocking. Conversely, mAbs with narrow or weak binding profiles, such as mAbs 107, 273, and 1444, showed little or no HBGA blocking capacity, consistent with their limited binding observed in ELISA. These results underscore the importance of integrating both binding and functional assays to identify antibodies with cross-reactive HBGA blocking potential and suggest that the most broadly reactive mAbs target conserved functional epitopes on the P domain.

### Publicness of norovirus mAbs

**Fig 7:**
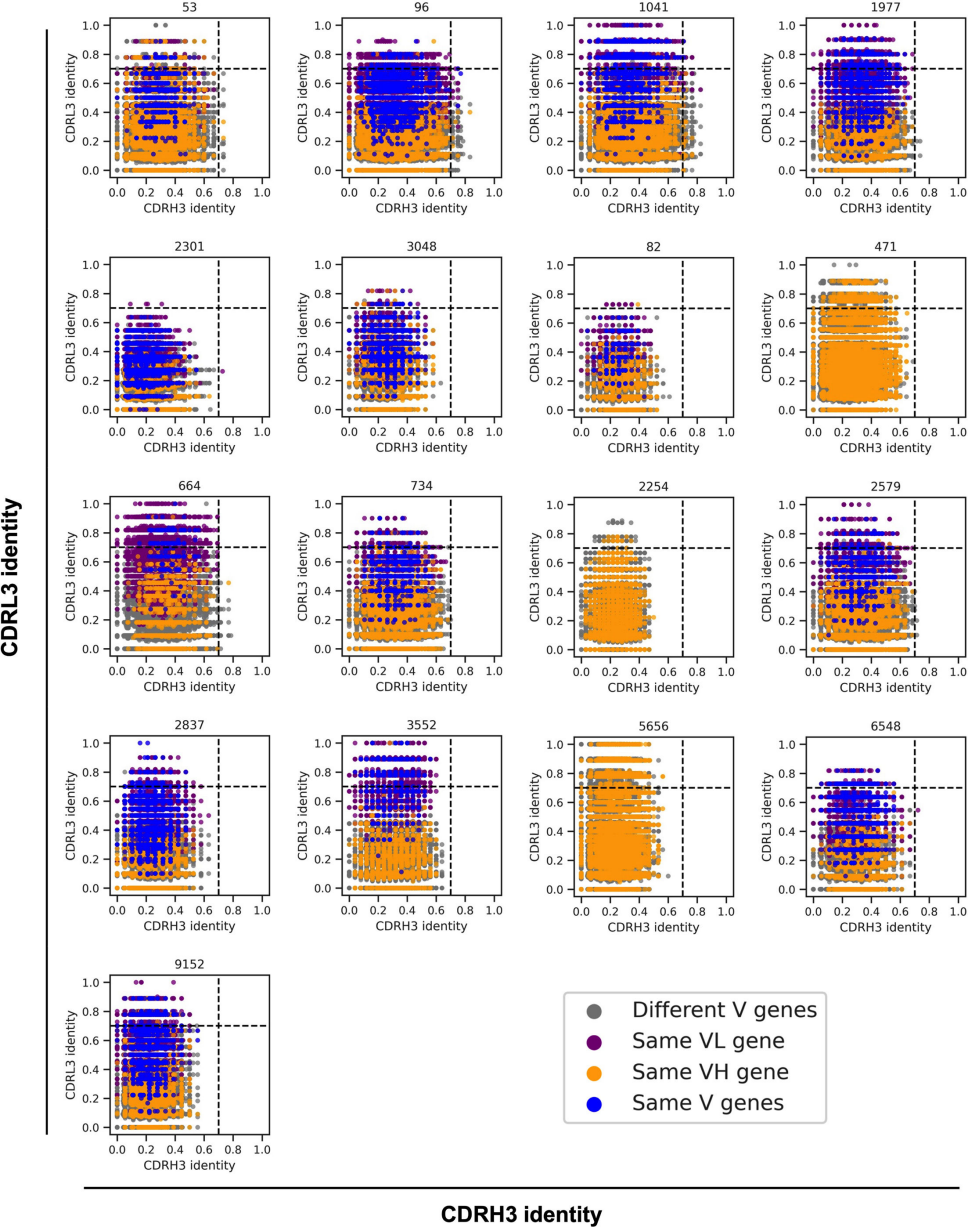
Comparison of norovirus-specific mAbs to published antibodies. Sequence similarity of lead antibody candidates to a public database ~1.7 million paired antibody sequences. Each point represents an antibody from the database being compared to the norovirus-specific mAb represented by the title for each panel, with the x-axis reflecting the amino acid sequence identity of the CDRH3, and y-axis the amino acid sequence identity of the CDRL3 being compared. Points are colored based on heavy and light V gene matches.

Sequences of the 17 norovirus mAbs that showed strong binding to one or more P domain (AUC > 4) were compared against a database of previously published antibodies consisting of 1,675,333 paired antibody sequences (47–53). Based on the gene usage and CDR3 similarity patterns (Table 1), norovirus mAbs were screened for publicness based on the given criteria: greater than 70% identity of CDRL3 and/or CDRH3, and same VH and/or VL gene usage (54). These criteria were chosen as previous work in our group established that shared IGHV:IGL(K)V gene usage in combination with a 70% amino acid identity threshold in the CDRH3 region in pairs of B cells to be sufficient for identifying public antibody clones (54). Of the 19 mAbs characterized, 16 had shared VH and VL gene pairings to antibodies within the public data set. Eight mAbs (3048, 82, 2254, 3552, 5656, 9152, 471, and 2837) did not share >70% CDRH3 sequence identity to any antibodies in the data set (Fig. 7; Table 2). All but two mAbs (96 and 1041) did not share CDRH3 and CDRL3 >70% to any antibody within the data set. Interestingly, mAb 1041 (which potently blocks GII.17) was the most public antibody identified, with >70% CDRH3 identity to 762 of the published antibodies and >70% CDHR3/CDRL3 identity to 30 of the published antibodies. Furthermore, mAbs 1041 had 32 corresponding full VH clones (defined as matching V gene usage with ≥0.7 CDR3 identity) and 2 corresponding full VH and VL clones (Fig. 7; Table 2). Withstanding mAb 1041, mAb 96 was the only other antibody with a corresponding full VH and VL clone identified in the data set. Interestingly, both public antibodies identified (1041 and 96) were broadly reactive across norovirus P domains and blocked HBGA binding to GII.10 to varied extents. Together, this analysis highlights that while most mAbs identified had some shared VH and VL gene pairings, they demonstrate unique patterns of somatic hypermutation, with few having CDHR3 and CDRL3 identity greater than 70%.

**TABLE 2:** Comparison of norovirus-specific mAbs to published antibodies^*a*^

Antibody ID	CDRH3 ≥0.7	CDRL3 ≥0.7	CDRH3 + CDRL3 ≥0.7	Same VH gene	Same VL gene	Same VH + VL gene	Full VH clones	Full VH + VL clones
53	17	11,329	0	71,389	27,177	973	2	0
96	179	25,043	4	69,391	96,210	2,971	7	1
1041	762	61,847	30	67,520	106,525	4,842	32	2
1064	4	722	0	60,847	89,946	4,154	0	0
1977	3	30,633	0	50,190	96,076	3,105	0	0
2301	1	4	0	163,292	88,640	10,541	0	0
3048	0	663	0	163,292	88,640	10,541	0	0
82	0	27	0	47,100	36,196	598	0	0
734	1	13,214	0	71,396	35,828	966	0	0
2254	0	455	0	91,038	0	0	0	0
2579	5	10,649	0	71,396	35,828	966	0	0
3552	0	34,573	0	47,845	50,149	1,957	0	0
5656	0	108,158	0	173,833	0	0	0	0
6548	1	6,667	0	41,700	175,326	5,998	0	0
9152	0	45,020	0	60,946	172,359	8,965	0	0
471	0	13,085	0	173,833	0	0	0	0
664	21	29,934	0	4,542	48,833	193	1	0
2837	0	8,879	0	163,292	88,640	10,541	0	0

^*a*^ Summary of counts based on comparisons shown in Fig. 7. Full clones are defined as having matching V gene usage with ≥0.7 CDR3 identity.

## DISCUSSION

Human noroviruses remain the leading cause of acute viral gastroenteritis worldwide, yet the immune correlates of protection and the breadth of naturally acquired antibody responses in the general population are still poorly defined. Despite frequent exposure throughout life (10, 55), individuals remain susceptible to repeated infection (56, 57), highlighting fundamental gaps in our understanding of norovirus immunity. In this study, we used LIBRA-seq to systematically map the norovirus-specific human antibody repertoire from healthy human donors, enabling high-resolution identification of mAbs with defined antigen specificity, cross-reactivity, and functional activity. This approach provides a snapshot of humoral immunity that complements prior studies focused on infected or vaccinated individuals.

A key finding of this study is the identification of multiple human mAbs capable of binding to genetically diverse norovirus GII genotypes, i.e., GII.4, GII.10, GII.17, GII.22, GII.23, GII.26, and GII.27. The binding patterns observed by ELISA closely mirrored predictions based on LIBRA-seq scores, validating the ability of this technology to accurately identify antigen-specific and cross-reactive B cells at scale. Functional characterization further revealed that a subset of these cross-reactive antibodies exhibited HBGA-blocking activity against GII.4 and GII.17. Notably, the two most broadly blocking antibodies identified in this study (mAbs 82 and 734) were isolated from the same donor, while no antibodies with similar breadth were recovered from the remaining donors. Given the limited number of donors analyzed and the selective criteria used for antibody expression, this finding likely reflects sampling depth rather than the true absence of broadly reactive responses in other individuals. Previous studies have shown that cross-genotype norovirus antibodies can be detected following natural exposure (29, 31), but such responses are often genotype specific (25–31).

The epitope mapping experiments provide additional insight into the antigenic landscape of the norovirus P domain. Competition ELISA data revealed at least three major epitope groups targeted by cross-reactive antibodies, with evidence for overlapping and partially distinct binding sites. This diversity of epitope recognition is notable, as it suggests that antibody breadth is not restricted to a single conserved region. From a vaccine design perspective, this finding is encouraging, as it implies that immunogens capable of presenting multiple conserved epitopes could elicit broader protective responses. Furthermore, public clonotype analysis demonstrates that most mAbs identified are rather unique, with only two norovirus-specific mAbs corresponding to full clones from the public antibody data set.

Our results extend previous work on norovirus nanobodies and mAbs targeting the capsid protein (25, 28, 32–39). We have shown that nanobodies and mAbs directly blocking the HBGA-binding pocket tend to be genotype restricted, whereas broadly reactive therapeutic nanobodies and mAbs often bind to conserved regions outside the HBGA-binding site, mainly at the lower region on the P domain. Consistent with this paradigm, many of the broadly reactive mAbs identified here did not necessarily exhibit the strongest HBGA-blocking activity. This supports the notion that norovirus HBGA blocking can occur through multiple mechanisms, including steric hindrance, virion aggregation, or interference with post-attachment steps of infection, rather than exclusively through direct blockade of HBGA engagement as was previously determined with nanobodies and other mAbs (25, 28, 32–39).

An important implication of these findings relates to the apparent paradox of frequent reinfection despite the presence of neutralizing mAbs (10, 55–57). Although broadly reactive mAbs exist in the general population, their abundance, durability, and functional potency may be insufficient to confer long-term immunity. Alternatively, protection may be strain specific and transient, with antigenic drift and genetic recombination enabling immune evasion over time (6). Our study does not directly address mAb longevity or protective thresholds, but it provides a framework for understanding how population-level antibody repertoires may influence susceptibility to emerging variants. Longitudinal studies linking mAb specificity and function to clinical protection will be essential to resolve these questions.

The use of LIBRA-seq represents a major strength of this study. Traditional approaches to mAb discovery often rely on memory B cells from recently infected or vaccinated individuals, which can bias results toward immunodominant or short-lived responses. In contrast, LIBRA-seq enables unbiased, high-throughput isolation of antigen specificity across diverse B cell populations, including rare cross-reactive clones. By applying this technology to healthy donors, we were able to capture mAbs likely generated through cumulative lifetime exposure rather than recent infection. This approach provides unique insight into the baseline humoral immunity present in the population and highlights the value of LIBRA-seq for studying complex, antigenically diverse pathogens. However, there are several limitations to consider. First, although we evaluated binding, HBGA blockade *in vitro* may not fully recapitulate protective immunity *in vivo*. Norovirus infection is influenced by host genetics, mucosal immunity, and viral factors beyond capsid-HBGA interactions. Second, the antigen panel, while diverse, does not encompass the full breadth of norovirus genotypes or emerging variants. Expanding antigen coverage may reveal additional cross-reactive or HBGA-blocking antibodies. Third, the use of isolated P domains may facilitate the identification of cross-genotype antibodies because the absence of the shell domain reduces steric constraints and may expose conserved epitopes that are less accessible on the assembled virion. However, antibodies identified using P domains may not always retain the same binding or HBGA blocking properties in the context of intact VLPs or infectious virus, where epitope accessibility can be restricted. In addition, antigen-specific B cells detected in PBMCs do not necessarily reflect circulating antibody repertoires, and the frequency of antigen-specific B cells may exceed the number of corresponding serum antibody clonotypes. These factors should be considered when interpreting the breadth and functional relevance of the antibodies identified in this study. Fourth, all mAbs were expressed as IgG1, which may not reflect the functional properties of their native isotypes, particularly IgA, which plays a critical role at mucosal surfaces. Last, the norovirus infection history of the blood donors was not considered in this study, preventing comparison between recent acute infections and long-term immunity.

Despite these limitations, our findings have important implications for norovirus vaccine and therapeutic development. The identification of conserved, functionally relevant epitopes recognized by naturally acquired human mAbs supports the feasibility of designing immunogens that elicit broad protection. Vaccines that focus immune responses toward these conserved regions, potentially through structure-guided antigen design or multivalent formulations, may overcome the challenge posed by norovirus diversity. In addition, the mAbs described here represent a new approach for the development and refinement of therapeutic antibodies and aid the refinement of other isolated neutralizing antibodies from patients previously infected with noroviruses with limited cross-reactivity (25–29). Ultimately, the key factor for developing effective therapeutic antibodies will be the breadth of cross-reactivity and finding conserved, vulnerable regions on the capsid.

Together, this study provides a comprehensive analysis of the human norovirus B cell repertoire using a high-throughput, antigen-specific single-cell sequencing approach. We demonstrate that broadly reactive and functionally active mAbs are present in healthy human donors and can target conserved epitopes across epidemiologically important norovirus genotypes. These findings advance our understanding of naturally acquired norovirus immunity and offer valuable insights to guide the rational design of next-generation vaccines and antibody-based interventions aimed at controlling this globally significant pathogen.

## MATERIALS AND METHODS

### Norovirus VLP and P expression

The norovirus VLPs were expressed as previously described (58). Briefly, the GII.4/Syd (JX459908), GII.10/Viet026 (AF504671), and GII.17/Kawa308 VLPs were purified using CsCl equilibrium gradient ultracentrifugation at 35,000 rpm for 18 h at 4°C (Beckman SW32.1 rotor). A distinct viral band was removed from the side of the tube with a syringe, and the VLPs were stored in PBS (pH 7.3) at 4°C. The integrity of the VLPs was confirmed by negative-stain EM, concentrated to 2–4 mg/mL, and stored at 4°C. The norovirus P domains were cloned and expressed as described (24). The P domain (GII.4/Syd, GII.10/Viet026, GII.17/CS-E1 [AY502009], GII.22 [AB083780], GII.23 [KT290889], GII.26 [KU306738], GII.27 [MG495077], and GX [MF373609]) was transformed into *E. coli* BL21 cells, which were grown in LB medium at 37°C. Expression was induced with 0.7 mM isopropyl β-d-1-thiogalactopyranoside (at OD_600_ = 0.6) for 18 h at 22°C. Cells were harvested by centrifugation and disrupted by sonication on ice. The His-tagged fusion-P domain protein was purified from a Ni-column (Qiagen) and digested with HRV-3C protease (Novagen) overnight at 4°C. The cleaved P domain was separated on the Ni-column and dialyzed in gel filtration buffer (GFB: 25 mM Tris-HCl pH 7.6 and 300 mM NaCl) overnight at 4°C. The P domain was further purified by size exclusion chromatography, concentrated to 2–4 mg/mL, and stored at 4°C.

### Sequence analysis

A phylogenetic tree of the norovirus P domain amino acid sequences was prepared using Clustal Omega (https://www.ebi.ac.uk/jdispatcher/msa/clustalo), and a multiple alignment of the partial P domain was produced using an online server (https://www.bioinformatics.org/sms/multi_align.html).

### Healthy donor peripheral blood mononuclear cell samples

PBMC donor samples were procured from StemCell Technologies catalog number 70025. Donor PBMC samples were collected between July 2022 and August 2024. All donors, except one, were male. Donors ranged in age from 20 to 39. Donors were screened and were negative for HIV-1, HIV-2, hepatitis B, and hepatitis C within 90 days of collection. Samples arrived frozen and were stored at −135°C until use.

### Biotinylation of antigens

Protein antigens were biotinylated using EZ-link Sulfo-NHS-Biotin No-Weigh kit (ThermoFisher) according to the manufacturer’s instructions. A 50:1 biotin-to-protein molar ratio was used for all reactions. Briefly, a 10 mM sulfo-NHS-Biotin solution was prepared, and the appropriate volume was added to each protein solution. Each reaction was allowed to proceed for 2 h on ice. Excess biotin reagent was removed using a Zebra Spin Desalting Column, prepared via manufacturer’s instructions.

### Oligonucleotide barcodes

We used oligos that possess a 15 base pair (bp) antigen barcode, a sequence capable of annealing to the template switch oligo that is part of the 10× bead-delivered oligos, and contain truncated TruSeq small RNA read 1 sequences in the following structure: 5′-CCTTGGCACCCGAGAATTCCANNNNNNNNNNNNNNNCCCATATAAGA*A*A-3′, where Ns represent the antigen barcode. Oligos were ordered from Sigma-Aldrich and IDT with a 5′ amino modification and HPLC purified. The following antigen barcodes were used: AACCTTCCGTCTAAG (GII.23), CTTCACTCTGTCAGG (GII.10), ACAATTTGTCTGCGA (GII.17), TGGTAACGACAGTCC (GII.27), GACCTCATTGTGAAT (GX), GACAAGTGATCTGCA (GII.26), CCGTCCTGATAGATG (GII.4), and ATTCGCCTTACGCAA (GII.22 and BG505-HIV envelope).

### Conjugation of oligonucleotide barcodes to antigens

For each antigen, a unique DNA barcode was directly conjugated to the antigen using a SoluLINK Protein-Oligonucleotide Conjugation kit (TriLink, S-9011) according to the manufacturer’s protocol. Briefly, the oligo and protein were desalted, and then the amino-oligo was modified with the 4FB crosslinker, and the biotinylated antigen protein was modified with S-HyNic. Next, the 4FB-oligo and the HyNic-antigen were mixed. This enables a covalent bond to form between the protein and the oligonucleotide. The concentration of the antigen-oligo conjugates was determined by a bicinchoninic acid (BCA) assay, and the HyNic molar substitution ratio of the antigen-oligo conjugates was analyzed using the NanoDrop according to the Solulink protocol guidelines. Excess oligonucleotide was removed from the protein-oligonucleotide conjugates using Superdex 200 Increase 10/300 GL on AKTA FPLC. Protein-oligonucleotide conjugates were verified with SDS-PAGE with Coomassie blue and silver stains. Aliquots of each labeled antigen were flash frozen and stored at –80°C for use in the secondary norovirus run. The optimal amount of antigen-oligonucleotide conjugates to use in antigen-specific B cell sorting was determined through titrations on Ramos B cells with known BCR specificity and healthy PBMCs.

### Enrichment of antigen-specific B cells

Healthy human PBMCs (StemCell Technologies) were thawed and resuspended in 10 mL of pre-warmed cRPMI. The cells were then washed with 10 mL of cRPMI at 300 rcf for 5 min at 4°C. Cells were counted, and viability was determined using Trypan Blue. Cells were resuspended in 2 mL staining buffer (dPBS +0.1% BSA) and stained with cell markers, including viability dye (ghost red 780), CD14-APCCy7, CD3-FITC, CD19-BV711, and IgG-PECy5 in the dark for 30 min at 4°C. Cells were then washed three times with staining buffer at 300 rcf for 5 min at 4°C. Antigen-oligo conjugates were then added and incubated with the cells for 30 min in the dark at 4°C. Cells were then washed three times with staining buffer at 300 rcf for 5 min at 4°C. Streptavidin-BV421 was added to label the cells with bound antigen and incubated for 15 min in the dark at 4°C. Cells were then washed three times with staining buffer, resuspended in staining buffer, and then sorted by FACS by the Vanderbilt Flow Cytometry Shared Resource core on a 5-laser FACS Aria III. Antigen-positive B cells were delivered to Vanderbilt Technologies for Advanced Genomics (VANTAGE) sequencing core at an appropriate target concentration for 10× Genomics library preparation and sequencing. FACS data were analyzed using FlowJo.

### 10× Genomics single-cell processing and next-generation sequencing

Single-cell suspensions were loaded onto the Chromium Controller microfluidics device (10× Genomics) and processed using previously described methods for LIBRA-seq. B cell Single Cell V(D)J solution was used for processing according to manufacturers’ suggestions for a target capture of 10,000 B cells per 1/8 10× cassette for B cells. Slight modifications were made to intercept, amplify, and purify the antigen barcode libraries as previously described.

### Sequence processing and bioinformatics analysis

Experiments were conducted using our established methodological pipeline, which takes paired-end FASTQ files of oligonucleotide libraries as input, to process and annotate reads for cell barcodes, unique molecular identifiers (UMIs), and antigen barcodes, resulting in a cell barcode-antigen barcode UMI count matrix (40). B cell receptor contigs were processed using CellRanger (10× Genomics) and GRCh38 as reference, while the antigen barcode libraries were also processed using CellRanger (10× Genomics). The cell barcodes that overlapped between the two libraries formed the basis of the subsequent analysis. Cell barcodes that had only non-functional heavy chain sequences, as well as cells with multiple functional heavy chain sequences and/or multiple functional light chain sequences, were eliminated, reasoning that these may be multiplets. We also aligned the B cell receptor contigs (filtered_contigs.fasta file output by CellRanger, 10× Genomics) to IMGT reference genes using HighV-Quest (53). The output of HighV-Quest was parsed using ChangeO and combined with an antigen barcode UMI count matrix. Finally, we determined the LIBRA-seq score for each antigen in the library for every cell as previously described. Across all eight sorts, we obtained 30,584 antigen-specific B cells after strict filtering criteria, including removal of B cells with no antigen UMI counts, unproductive VDJ regions (Variable [V], Diversity [D], and Joining [J] gene segments), and B cells with *N* > 1 (which indicates more than one heavy chain) which were down-selected based on LSS.

### Selection of B cells for monoclonal antibody expression

B cells identified by LIBRA-seq were selected for mAb expression using predefined criteria intended to enrich for diverse binding cross-reactivity profiles while maintaining sequence diversity. Selection was not based on the total number of antigen-reactive B cells detected per donor. Instead, candidate cells were prioritized if they met the following criteria: (i) LSS > 2 for at least two norovirus antigens, (ii) low LSS for the negative control antigen (HIV envelope protein), (iii) predicted binding to multiple norovirus genotypes, (iv) diverse inferred germline gene usage, (v) variation in native isotype, (vi) evidence of somatic hypermutation, and (vii) differences in CDR length. Because all criteria were applied together, the number of B cells selected from each donor varied and was not proportional to the total number of reactive cells recovered. In some donors, many B cells showed limited predicted breadth, redundant sequences, or did not pass sequence quality filtering and were therefore not considered further. Donor serum reactivity was not used as a selection criterion, as LIBRA-seq identifies antigen-specific B cells at the single-cell level independently of bulk serum responses. Based on these criteria, 25 B cells representing distinct predicted lineages were selected for recombinant expression. All antibodies were expressed as human IgG1 regardless of native isotype and purified in PBS for further analysis.

### Antibody and nanobody recombination production

Heavy and light chain genes of antibodies selected for further characterization were inserted into plasmids (pcDNA3.1) that encode the constant region for the human IgG1 heavy chain and respective lambda and kappa light chains. Antibodies were initially expressed with Genscript using TurboCHO 2.0 cells, followed by Protein A purification and SEC-HPLC. Antibodies were re-expressed in-house using Expi293F cells to increase stock. Cells were maintained at 8% CO_2_ and 37°C with shaking in Freestyle F17 Expression Media (Thermo Fisher) supplemented to a final concentration of 0.1% Pluronic Acid F-68 and 4 mM L-glutamine. Cells were co-transfected with the heavy and light chain plasmids using Expefectamine transfection reagent, and cells were cultured for 4–5 days. Cells were harvested by centrifugation at 4,000 rcf for 20 min, and the supernatant was filtered with 0.45 μm Nalgene Rapid Flow Disposable Filter Units with PES membrane. The resulting supernatant was run over a protein A column. The column was washed with PBS, and antibodies were eluted with 100 mM Glycine HCl at 2.7 pH directly into a 1:10 volume of 1M Tris-HCl pH 8.0. Eluted antibodies were buffer exchanged three times into PBS using Amicon Ultra centrifugal filter units (30 kDa). Antibodies were concentrated to 1 mg/mL and analyzed by SDS-PAGE. Nanobodies used for controls in HBGA blocking studies were produced as described earlier (38, 39).

### Direct ELISA

A direct ELISA was conducted to evaluate the binding of LIBRA-seq-identified antibodies to their predicted antigen(s). Soluble, purified antigen was plated at a concentration of 2 µg/mL on 384-Well Polystyrene Plates (Thermo Scientific) and incubated overnight at 4°C. The next day, plates were washed three times with PBS supplemented with 0.05% Tween20 in PBS (PBS-T) and blocked with 5% BSA in PBS-T. Plates were incubated at room temperature for 2 h and then washed three times with PBS-T. Primary antibodies were diluted in 1% BSA in PBS-T, starting at a concentration of 10 μg/mL with a serial 1:5 dilution, then added to the plate. Plates were incubated for 1 h at room temperature and then washed three times with PBS-T. The secondary antibody, goat anti-human IgG conjugated to peroxidase, was added at a dilution of 1:10,000 in 1% BSA in PBS-T to the plates and then incubated for 1 h at room temperature. Plates were washed three times with PBS-T and developed with the addition of TMB substrate. Plates were incubated at room temperature in the dark for 10 min before the reaction was stopped with 1 N sulfuric acid. Plates were read at 450 nm (OD₄₅₀). The data shown are from one biological replicate. Each ELISA was repeated minimally in biological and technical duplicate. The AUC was calculated using GraphPad Prism Version 10.6.1.

### Antibody-antibody competition ELISA

Wells of a 96-well microtiter plate were coated with 100 μL of 2 μg/mL GII.17 P domain protein overnight at 4°C. Plates were blocked with 200 μL of 5% BSA in PBS-T for 1 h before washing three times with 0.1% Tween-20 in PBS (PBS-T). Primary mAbs were added to wells (80 μL/well) in duplicate and incubated for 1 h at room temperature, with the final concentration of each mAb at 10 μg/mL. A biotinylated preparation of recombinantly produced mAb was added to wells of each primary mAb at an initial concentration of 5 μg/mL in a volume of 20 μL/well (for a final concentration of 1 μg/mL in each well), without washing of the unlabeled antibody. Plates were spun at 800 × *g* for 1 min and then incubated for 1 h at room temperature. Plates were washed three times with PBS-T and then 100 μL of horseradish peroxidase-conjugated anti-biotin antibody was added to each well at a concentration of 1:4,000 and incubated for 1 h at room temperature. Plates were washed three times with PBS-T and developed with the addition of TMB substrate. Plates were incubated at room temperature in the dark for 10 min before the reaction was stopped with 1 N sulfuric acid and plates measured (OD₄₅₀). The signal obtained for binding of the biotin-labeled mAb in the presence of the primary mAb was expressed as a percentage of the binding of the reference antibody alone after subtracting the background signal. Tested mAbs were considered competing if their presence reduced the reference antibody binding to less than 40% of its maximal binding and non-competing if the signal was greater than 71%. A level of 41% to 70% was considered intermediate competition. The data shown are from one biological replicate.

### HBGA blocking assay

The HBGA blocking assay was performed as previously described with minor modifications (59). Saliva from a blood type A donor was heated at 95°C for 10 min, briefly centrifuged, and the supernatant was stored at 4°C until use. Saliva was diluted 1:1,000 in PBS, and 100 µL was added to triplicate wells of a microplate and incubated overnight at 4°C. Plates were washed three times with PBS-T and blocked with 300 µL of 5% skim milk in PBS for 1 h at room temperature. In a deep-well plate, mAbs were initially diluted to 50 µg/mL in PBS and then subjected to twofold serial dilutions. VLPs were diluted to 5 µg/mL and mixed 1:1 with the mAbs, yielding a starting mAb concentration of 25 µg/mL. The VLP-mAb mixtures were incubated for 1 h at room temperature. Subsequently, 100 µL of each mixture was added to triplicate wells and incubated for 1 h at 37°C. Plates were washed and incubated with either GII.4 or GII.17 rabbit polyclonal antibody (100 µL, 1:20,000 dilution in PBS) for 1 h at 37°C. Following washing, a secondary antibody HRP-conjugated goat anti-rabbit IgG (100 µL, 1:40,000 dilution in PBS) was added and incubated for 1 h at room temperature. After washing, plates were developed with 50 µL OptEIA TMB substrate in the dark for 30 min at room temperature. The reaction was stopped with 25 µL of 1 N HCl, and absorbance was measured (OD₄₅₀). Negative control wells (no inhibitor and no secondary antibody) were subtracted from all OD₄₅₀ values, and untreated VLPs were set as 100% binding. Inhibition (%) was calculated as: 1 – (mean OD₄₅₀ treated/mean OD₄₅₀ reference) × 100 (59, 60). IC_50_ values were calculated using nonlinear regression (four-parameter logistic curve, X is concentration) using GraphPad Prism Version 10.6.1.

### Public clonotype analysis

To place the molecular features of the antibodies in context, we performed a public clonotype analysis in which the norovirus-specific mAbs were compared with a database of 1,675,333 previously reported paired antibody sequences. This database consists of a previously curated data set (47), expanded with antibodies from public databases (48–53). Antibodies were considered related based on similarity in V-gene usage and CDR3 sequence identity, which are commonly used measures to assess clonal relationships and repertoire convergence. CDR3 identity was calculated using the Levenshtein distance metric, and antibodies sharing ≥70% CDRH3 amino-acid identity were considered to be public, a threshold frequently used in human B cell repertoire studies to identify antibodies derived from similar B cell lineages. Scatterplots in Fig. 7 show pairwise comparisons of antibodies based on heavy-chain (CDRH3) and light-chain (CDRL3) identity, with color coding indicating whether antibody pairs share VH genes, VL genes, both, or neither. This analysis allows assessment of whether the antibodies identified here resemble previously described public antibody responses or represent distinct repertoire features.

## Data Availability

The sequences for antibodies identified and characterized in this study have been deposited to GenBank under GenBank accession numbers PZ268779 to PZ268828. Materials can be provided by Ivelin S. Georgiev and Grant S. Hansman pending scientific review and a completed material transfer agreement.

## SUPPLEMENTAL MATERIAL

Fig. S1 jvi.00497-26-s0001.eps LSS heat maps for B cells with LSS >2 for 1+ norovirus antigen.

ASM does not own the copyrights to Supplemental Material that may be linked to, or accessed through, an article. The authors have granted ASM a non-exclusive, world-wide license to publish the Supplemental Material files. Please contact the corresponding author directly for reuse.
